# HOS1-mediated both ubiquitination and nuclear-import are crucial to nitrate signaling

**DOI:** 10.3389/fmolb.2026.1752969

**Published:** 2026-03-18

**Authors:** Lin-Bei Xie, Xin-Yue Yang, Zhong-Wei Zhang, Xiao-Yan Tang, Shu Yuan

**Affiliations:** International Science and Technology Cooperation Base for Efficient Utilization of Nutrient Resources and Fertilizer Innovation, College of Resources, Sichuan Agricultural University, Chengdu, China

**Keywords:** HOS1, nitrate depletion, NLP7, nuclear-import, SnRK1, ubiquitination

## Introduction

Acting as central energy sensors, the evolutionarily conserved sucrose non-fermenting-1-related (SnRK1) proteins in plants are functional homologs of yeast Sucrose Non-Fermenting 1 (SNF1) and mammalian adenosine monophosphate-activated protein kinase (AMPK). This kinase family is essential for sustaining cellular energy balance and coordinating processes related to growth, development, and stress responses (Baena-González et al., 2007; Belda-Palazón et al., 2020; Broeckx et al., 2016; Feng et al., 2025; Wang et al., 2021; Williams et al., 2014; Yuan et al., 2016). In Arabidopsis, the functional SnRK1 complex is a heterotrimer, consisting of a catalytic α-subunit (either SnRK1α1/KIN10 or SnRK1α2/KIN11) along with regulatory β and βγ subunits (Baena-González et al., 2007; Fragoso et al., 2009; Zacharaki et al., 2022). A key mechanism of SnRK1 involves the direct phosphorylation of developmental regulators. For instance, under low nitrate conditions, KIN10 phosphorylates NIN-like protein 7 (NLP7), a primary transcription factor in nitrate signaling, which triggers its degradation and consequently limits plant growth (Wang et al., 2022).

The high expression of osmotically responsive gene 1 (HOS1) is an E3 ubiquitin ligase with a broad regulatory scope. While it is recognized for mediating the ubiquitination of CBF expression 1 (ICE1), homeodomain-leucine zipper protein 1 (HAT1) and CONSTANS (CO) to control cold signaling and flowering, its functions extend to promoting thermo-tolerance and other stress tolerance through the regulation of flowering locus C (FLC) chromatin, phytochrome interacting factor 4 (PIF4) transcription, and DNA helicase RecQ2 expression (Dong et al., 2006; Han et al., 2020; Ishitani et al., 1998; Jung et al., 2013; Kang et al., 2025; Kim et al., 2017; Lazaro et al., 2012; Lazaro et al., 2015; Lee et al., 2001).

In Arabidopsis, HOS1 is a unique protein that contains a domain homologous to nucleoporins (NUPs) found in yeast and animals (Jung et al., 2014). Its function at the nuclear pore complex (NPC) is supported by direct interactions with components NUP96 and NUP160, as well as associations with RAE1 (RNA export factor 1), NUP43, and NUP85 (Cheng et al., 2020; Li et al., 2020; Sanchez Carrillo et al., 2025; Tamura et al., 2010; Zhu et al., 2017). This localization suggests HOS1 helps regulate nucleo-cytoplasmic trafficking. Consistent with this role, mutants of HOS1 and other NUPs share similar phenotypes, including defects in mRNA export, protein trafficking, temperature signaling, circadian rhythms, and flowering time (MacGregor et al., 2013; Zhang et al., 2020). One specific consequence in the *hos1* mutant is the reduced nuclear accumulation of the PIF4 transcription factor, which impairs the plant’s response to warm temperatures (Zhang et al., 2020). Recently, HOS1 has been shown to play an essential role in specific meiotic function, anchoring and positioning telomeres to facilitate the pairing of homologous chromosomes (Fernández-Jiménez et al., 2023).

Although nuclear localization of glyceraldehyde-3-phosphate dehydrogenase 1 is dependent on its ubiquitination by an E3 ubiquitin-protein ligase (Peralta et al., 2016), ubiquitination may not be involved in most protein nuclear transports. The ubiquitination function and the nuclear import activity of HOS1 may be independent of each other.

## HOS1 is invlovled in both the nuclear import and the ubiquitin-mediated degradation of SnRK1

According to Margalha et al. (2023), HOS1 modulates the nuclear abundance of SnRK1α1 during low-energy stress without affecting its overall protein levels. In contrast, a recent investigation by Liu et al. (2026) found that HOS1 facilitates the degradation of SnRK1α1 via its E3 ligase function under nitrate treatment. Margalha et al. (2023) used prolonged dark stress treatments (2–7 days); while Liu et al. (2026) adopted 5 mM KNO_3_ or 5 mM KCl treatment in nitrate-free media. Nevertheless, the discrepancy may not be only attributed to the difference in stress conditions. This inconsistency, which was not thoroughly addressed by Liu et al. (2026), might be clarified by separately assessing SnRK1 degradation rates in the cytoplasm and nucleus. The nuclear localization of HOS1 is well-established, as confirmed by Lazaro et al. (2012) using the bimolecular fluorescence complementation (BiFC) assays and is consistent with the nuclear presence of its targets like CO (Dong et al., 2006; Valverde et al., 2004). Given that the majority of KIN10 is cytoplasmic (Margalha et al., 2023; Yuan et al., 2016), other cytoplasmic degradation mechanisms are likely involved. Potential candidates include SIZ1, a SUMO (Small Ubiquitin-like Modifier) E3 ligase that targets SnRK1 for proteasomal degradation (Crozet et al., 2016), and SKP1, a conserved SCF (Skp1-cullin-F-box) ubiquitin ligase subunit, which interacts with the pleiotropic regulatory locus 1 (PRL1)-binding C-terminal domains of SnRKs to promote their proteolysis (Farrás et al., 2001). Further research is needed to determine if SIZ1-mediated SUMOylation or SKP1-mediated ubiquitination contributes to the nitrate-induced degradation of cytosolic KIN10.

**FIGURE 1:**
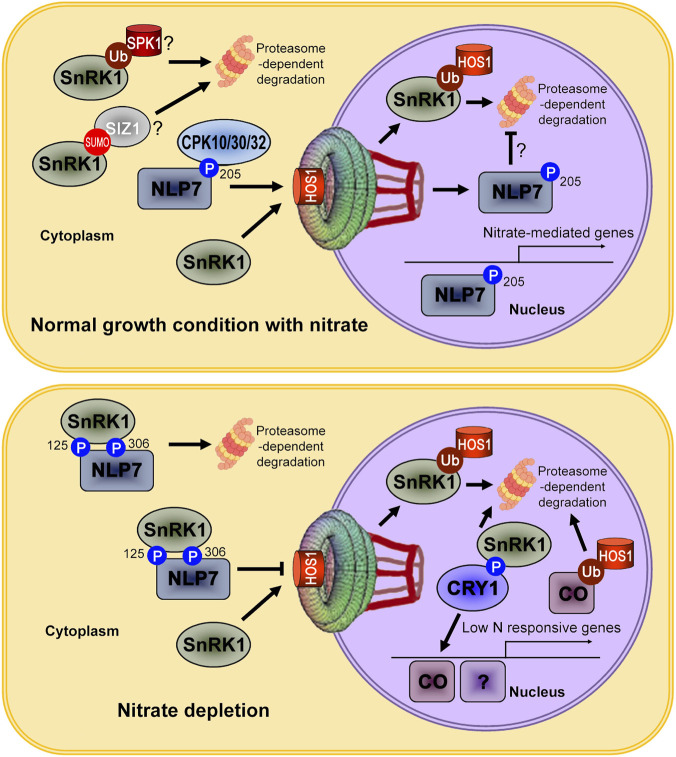
HOS1 mediates ubiquitination and nuclear-import in nitrate signaling. Nitrate triggers a Ca^2+^ signal leading to the phosphorylation of NLP7 at ser-205 by Ca^2+^-sensor protein kinases CPK10, 30 and 32, which moves NLP7 to the nucleus to stimulate growth. However under nitrate depletion, a different kinase, SnRK1, phosphorylates NLP7 at ser-125 and ser-306. This modification traps NLP7 in the cytoplasm for degradation, effectively turning off the nitrate-responsive growth program. HOS1 has a dual function, participating in both the nuclear import and the ubiquitin-mediated degradation of SnRK1 in the nucleus. HOS1 may also participate in the nuclear import of NLP7. Given that the majority of SnRK1 is cytoplasmic, other cytoplasmic degradation mechanisms are likely involved. For examples, SIZ1-mediated SUMOylation and SKP1-mediated ubiquitination may contribute to the nitrate-induced degradation of cytosolic SnRK1. The phosphorylation at Ser-205 may protect NLP7 from ubiquitination by HOS1 or other nuclear ubiquitin ligases, thereby preventing its rapid turnover. Nitrate depletion also promotes SnRK1 activity and declines the abundance of nuclear CRY1 via phosphorylation. CRY1, in turn, functions as an input to modulate the phase and amplitude of the circadian clock, as well as flowering-time genes like CO. Since HOS1 is known to mediate the ubiquitination and degradation of CO in the nucleus, it may play an indirect role in regulating flowering genes induced by nitrogen deficiency. There might be some other transcription factors responsive to nitrate depletion.

Margalha et al. (2023) demonstrated that nuclear SnRK1 accumulation is impaired in the *hos1* mutant, a finding that contradicts the expectation if HOS1 was only responsible for SnRK1 degradation in the nucleus. Consequently, they posit that HOS1 is necessary for the nuclear transport of SnRK1. We extend this model by speculating that HOS1 has a dual function, participating in both the nuclear import and the ubiquitin-mediated degradation of SnRK1 (Figure 1).

## HOS1 may destabilize NLP7 indirectly or regulate NLP7 nuclear import directly

NLP7 functions as a central regulatory switch for plant growth, with its activity determined by nutrient-dependent phosphorylation (Fu et al., 2022; Liu et al., 2017; Liu et al., 2022). Liu et al. (2017) showed that nitrate triggers a Ca^2+^ signal leading to the phosphorylation of NLP7 at ser-205 by Ca^2+^-sensor protein kinases CPK10, 30 and 32, which moves NLP7 to the nucleus to stimulate growth. However, Wang et al. (2022) demonstrated that under energy stresses (carbon deficiency or nitrate depletion), a different kinase, KIN10, phosphorylates NLP7 at ser-125 and ser-306. This modification traps NLP7 in the cytoplasm for degradation, effectively turning off the nitrate-responsive growth program (Figure 1).

NLP7 protein levels are significantly reduced in *hos1* mutants. According to Liu et al. (2026), HOS1 may stabilize the NLP7 protein by facilitating the degradation of KIN10 in the presence of nitrate. This is complicated by the finding that KIN10 itself promotes the phosphorylation and degradation of NLP7, but this regulation is confined to the cytoplasm and only happens during nitrate depletion (Wang et al., 2022). One potential explanation is that a HOS1 mutation could lead to an accumulation of cytosolic SnRK1, which would then indirectly destabilize NLP7. Another possibility is that HOS1 directly assists in the nuclear import of NLP7; in the *hos1* mutant, NLP7’s retention in the cytoplasm could make it more susceptible to degradation (Figure 1). Future experiments are required to distinguish between these two models. Measuring both the steady-state level and the degradation rate of NLP7 in wild-type and *hos1* mutants under nitrate-depleted conditions could clarify the mechanism. Since NLP7 remains predominantly cytosolic under nitrate depletion, the presence or absence of nuclear HOS1 should have minimal impact on the turnover of cytosolic NLP7 in this context. In contrast, under nitrate supply, NLP7 must be phosphorylated at Ser-205 and imported into the nucleus to function. It is possible that this specific phosphorylation protects NLP7 from ubiquitination by HOS1 or other nuclear ubiquitin ligases, thereby preventing its rapid turnover, a hypothesis that also warrants future investigation.

## HOS1 may mediate ubiquitination and degradation of CO under nitrogen deficiency

Under low nitrogen availability, the increased ferredoxin NADP^+^ oxidoreductase (FNR1) activity and NADPH/NADP^+^ and ATP/AMP ratios modulate SnRK1 activity (Yuan et al., 2016). This same study also demonstrated that AMPK activity and its nuclear localization exhibit a rhythmic pattern, which is inversely related to the abundance of nuclear cryptochrome 1 (CRY1) protein. CRY1, in turn, functions as an input to modulate the phase and amplitude of the circadian clock (Zhang et al., 2019; Zhou et al., 2016). Yuan et al. (2016) further reported that low nitrogen levels upregulate, while high nitrogen levels downregulate, the expression of core clock components such as LHY (Late Elongated Hypocotyl), CCA1 (Circadian Clock Associated 1) and TOC1 (Timing Of CAB expression 1), as well as flowering-time genes like GIGANTEA (GI) and CO. Since HOS1 is known to mediate the ubiquitination and degradation of CO in the nucleus (Lazaro et al., 2012), it may play an indirect role in regulating flowering genes induced by nitrogen deficiency (Figure 1). The identity of other transcription factors responsive to nitrate depletion remains an area for future discovery.

## Conclusion

In summary, HOS1 appears to function both in the nuclear ubiquitination and degradation of SnRK1α1 and CO, and as an essential factor for the nuclear import of SnRK1α1 and NLP7. NLP7 and CO are key transcription factors for nitrate signaling and nitrogen deficiency signaling, respectively, which broadly regulate nuclear gene expression to control growth and development in response to nitrogen availability.
